# Study on the Co-Benefits of Air Pollution Control and Carbon Reduction in the Yellow River Basin: An Assessment Based on a Spatial Econometric Model

**DOI:** 10.3390/ijerph19084537

**Published:** 2022-04-09

**Authors:** Zhongyao Cai, Xiaohui Yang, Huaxing Lin, Xinyu Yang, Ping Jiang

**Affiliations:** 1 Department of Environmental Science & Engineering, Fudan University, Shanghai 200433, China; 21210740113@m.fudan.edu.cn (Z.C.); 20210740111@fudan.edu.cn (X.Y.); 20110740039@fudan.edu.cn (H.L.); yangxinyu2975@163.com (X.Y.); 2 Fudan Tyndall Center, Fudan University, Shanghai 200433, China

**Keywords:** carbon reduction, air pollution control, co-benefits, spatial spillover effects, DPSIRM, Yellow River Basin

## Abstract

To assess the green and low-carbon development of the Yellow River Basin (YRB) in China, this study utilizes an evaluation index system based on the framework of driving force, pressure, state, impacts, response, and management, and it measured the comprehensive scores of the co-benefits of carbon reduction and air pollution control in the YRB. The global Moran index was used to analyze the spatial correlation characteristics of co-benefits, and a generalized spatial measurement model was constructed to demonstrate their spatial spillover effects. The results show that the co-benefits steadily increased every year. The co-benefits had a significant positive spatial correlation and showed a development trend of “up–down–up”. According to the spillover effect test, the economic development level, education level, and intensity of environmental regulations had significant positive effects, while the level of urbanization and foreign investment had significant negative effects. Considering these results and the aim of promoting green and low-carbon development, clear detection of the spatial spillover characteristics of the co-benefits should be prioritized, followed by an understanding of the spatial transmission mechanism of carbon and air pollutant emission and transfer. Policy recommendations are also proposed including upgrading industrial structure, focusing on the development of modern services and high-tech industry, and strictly implementing the industrial environment access system.

## 1. Introduction

The concentrations of greenhouse gases (GHGs) are increasing, leading to changes in average temperatures, precipitation, and the frequency of extreme weather events; in addition, climate change, which is mainly caused by human activities, is becoming one of the greatest global challenges of the 21st century [1]. Concurrently, air pollution threatens human health and hinders the sustainable development of the economy and society. This has also become one of the most arduous challenges for society. According to data released by China’s Ministry of Ecology and Environment in 2018, the compliance rate of China’s urban environmental air quality was only 35.8%, indicating that air pollution problems in many regions of China must be urgently resolved. The latest version of the “Law of the People’s Republic of China on the Prevention and Control of Air Pollution” clearly states that coordinated control of air pollutants and GHGs should be implemented. The solution to regional air pollution and climate change issues and the improvement of air quality are inseparable from the coordinated and unified response strategies for carbon reduction and air pollution control.

In September 2019, the Chinese government incorporated “ecological protection and high-quality development of the Yellow River Basin (YRB)” into the national strategy for regional coordination [2]. The YRB spans nine provinces in three major regions: west, middle, and East China. It had a total permanent population of 420 million in 2020, accounting for approximately 30.1% of China’s total population. In 2020, the regional gross domestic product (GDP) was 25.4 trillion yuan, accounting for approximately 25.0% of China’s regional GDP. The YRB plays a vital role in promoting national ecological protection and economic and social development [3]. However, the dense population and thriving secondary industry in the YRB leads to the emission of large amounts of GHGs and air pollutants. This has caused increasingly serious environmental pollution, affecting the self-purification ability of the ecosystem and greatly restricting the coordinated development of the regional economy and ecological environment [4,5].

With the emergence of climate change and air pollution awareness, the academic community has deepened research on carbon reduction and air pollution control. Specifically, research on the co-benefits of carbon reduction and air pollutant control has attracted increasing attention from researchers worldwide [6,7,8]. China’s Ministry of Environmental Protection defined co-benefits as reducing greenhouse gas (GHG) emissions and other regional pollutant emissions, while controlling regional pollutant emissions and ecological construction, reducing carbon dioxide, and other GHG emissions or absorbing a part of GHGs [9]. The co-benefits discussed in this paper refer to the achievement of mitigating climate change, solving local environmental and developmental problems as well as improving public health through the implementation of energy and environmental protection policies [10]. “Co-benefits” is a term that includes two layers of implications. One refers to the narrow co-benefits, which means the immediate benefits achieved (e.g., reducing GHG and pollutants emissions) because of the implementation of energy, climate, and air pollution policies [11,12,13,14]. Another term of co-benefits is defined as a generalized concept with the broad co-benefits, which refers to achieving environmentally sustainable development as the premise, while maintaining both economically and socially sustainable development at the same time [15,16]. Studies have put more attention on the narrow definition of co-benefits so far [17,18,19,20]. Many of these studies combine policy scenario analysis and model quantitative estimation, measuring and comparing the co-benefits under different policy combinations via pollutant concentrations and emissions [21,22,23,24,25]. Foreign scholars mainly study the co-benefits of carbon reduction [26,27,28,29,30], while domestic scholars focus on the co-benefits of air pollution control [31,32,33,34,35]. However, in recent years, domestic scholars have paid increasing attention to carbon reduction [36,37].

In China and abroad, the main research fields of co-benefits are energy, transportation, and industry. The main research subjects are the world, a certain country, region, or sector, while research on a certain region or urban agglomeration is less common [38]. Considering that economic development, scientific and technological progress, government policy regulation, etc., directly or indirectly affect the environmental and economic benefits of a region and even a country, this study attempted to explore the broad co-benefits. Based on the coordination of carbon reduction and air pollutant control, this study evaluated the co-benefits of carbon reduction and air pollution control in the YRB and explored the spatial spillover effects by integrating macro indicators such as economy, population, and government policies.

## 2. Methodology

### 2.1. Evaluation Model

To objectively evaluate the co-benefits of carbon reduction and air pollution control in the YRB, the entropy weight method was used to provide objective weights via an established evaluation index system based on the framework of driving force, pressure, state, impacts, response, and management (DPSIRM). The driving force (D) is determined by the economic development level, industrial structure, population size, and level of scientific and technological innovation, which results in energy consumption pressure (P). Energy consumption intensity directly affects the emission level of air pollutants and GHGs (S), which then influences the impacts (I) of air pollution and the greenhouse effect. In response (R) to these impacts, the government will adopt a series of policies and measures. Finally, the government will take corresponding action to implement better environmental management (M). The results from management changes will further affect the driving force, pressure, state, impacts, and response in the system of the co-benefits and form a two-way circular feedback mechanism. The quantity of information transmitted to decision makers determines the objective weight and is expressed by entropy. The smaller the entropy value, the more information the index contains and transmits, and the greater the corresponding weight. This method avoids the disadvantage that the weight assigned by traditional methods, such as the expert scoring method, is too subjective.

First, the data are standardized, assuming that k indicators are defined as $X_{1},X_{2},\cdots ,X_{k}$, where $Xi=\left|x_{1},x_{2},\cdots ,x_{n}\right|$. Assuming that the standardized value of each index is $Y_{1}^{},Y_{2},\cdots ,Y_{k}$, $Y_{ij}=\frac{X_{ij}-\min(X_{i})}{\max(X_{i})-\min(X_{i})}$. The information entropy of each index is $E_{j}$, as shown in Formula (1), where $p_{ij}=\frac{Y_{ij}}{\sum_{i=1}^{n}Y_{ij}}$. If $p_{ij}=0$, then $\lim_{pij-0}p_{ij}\ln p_{ij}=0$ is defined. The information entropy of each index is calculated as $E_{1},E_{2},\cdots ,E_{k}$, and the weight of each index is calculated as $W_{j}$ as shown in Formula (2). In Formula (3), Yi is the final score [39].

(1) $$E_{j}=-\ln{\left(n\right)}^{-1}\sum_{i=1}^{n}p_{ij}\ln p_{ij}$$

(2) $$W_{j}=\frac{1-E_{j}}{k-\sum E_{j}}(j=1,2,\cdots ,k)\;$$

(3) $$Y_{i}=\sum_{j=1}^{n}Y_{ij}W_{j}$$

### 2.2. Global Moran Index

This paper describes the global spatial autocorrelation of the global Moran index to test whether there is spatial correlation and heterogeneity in the co-benefits of carbon reduction and air pollution control in the YRB. This is also the first in-depth study of the spatial spillover effects. The spatial spillover effects refer to the impact that an activity in an area will not only produce the desired effect in the area, but also have an impact on people or society outside the area [40]. The calculation of the global Moran index is shown in Formula (4), where $Y_{i}$ and $Y_{j}$ represent the comprehensive scores of the co-benefits of carbon reduction and air pollution control in the i and j regions of the YRB, n is the number of research objects, $\bar{Y}$ is the average of the comprehensive scores, and $W_{ij}$ is the spatial weight matrix.

In Formula (5), Z is the standard normal statistic constructed using the global Moran index. When the value Z is significantly positive, it indicates that there is a positive spatial autocorrelation between the co-benefits of carbon reduction and air pollution control; that is, similar co-benefits of carbon reduction and air pollution control indicate spatial agglomeration. When the value Z is significantly negative, it indicates that there is a negative spatial autocorrelation between the co-benefits of carbon reduction and air pollution control; that is, the similar co-benefits of carbon reduction and air pollution control indicate spatial dispersion. When the value Z is zero, it shows that the co-benefits of carbon reduction and air pollution control present a random spatial distribution [41,42].

(4) $$I=\sum_{i=1}^{n}\sum_{j=1}^{n}W_{ij}(Y_{i}-\bar{Y})(Y_{j}-\bar{Y})/\sum_{i=1}^{n}\left(Y_{i}-\bar{Y})(Y_{i}-\bar{Y}\right)$$

(5) $$Z=(I-E(I))/\sqrt{Var(I)}\;$$

### 2.3. Spatial Econometric Model

Panel spatial econometric models are generally divided into spatial lag and spatial error models. The former focuses on spatial lag dependence, while the latter focuses on spatial error dependence. Formula (6) represents the spatial lag model, while Formula (7) represents the spatial error model [43]. The adjacent-space matrix was used to represent the spatial association pattern. This study used the spatial autocorrelation (SAC) model, which includes both spatial lag and spatial error to analyze the factors influencing the co-benefits and does not set other constraints. $Y_{k,t}$ represents the co-benefits of carbon reduction and air pollution control in the k region at time t. W denotes the spatial weight matrix. X is the explanatory variable, while α, η, γ, and λ are the regression coefficients. Finally, u and ε are the disturbance terms [44].

(6) $$\ln Y_{k,t}=\alpha +\eta W\ln Y_{k,t}+\gamma \ln X_{k,t}+u_{k,t}$$

(7) $$u_{k,t}=\lambda Wu_{k,t}+\varepsilon_{k,t}$$

## 3. Empirical Estimation

### 3.1. Evaluation of the Co-Benefits of Carbon Reduction and Air Pollution Control

Considering the differences in the emission levels of carbon and air pollutants and the related policies in time and space, this study comprehensively evaluated the co-benefits of carbon reduction and air pollution control in the YRB by integrating the macro factors of society, economy, and environment. Following the premise of comprehensiveness, objectivity, systematicness, and operability as well as referring to the research work of Qiao et al. [45], the evaluation index system was constructed based on the DPSIRM framework, and the weight was determined by the entropy weight method presented in Section 2.1, as shown in Table 1.

Combined with the relevant data from the China Statistical Yearbook, China Environmental Statistical Yearbook, and the provincial statistical yearbooks, the comprehensive scores of the co-benefits of carbon reduction and air pollution control in the YRB were obtained after empowerment and differentiation as shown in Table 2.

### 3.2. Spatial Correlation Analysis

The inter-regional transfer trend of high energy consumption, high-pollution, and high-emission industries in the YRB is prominent. Existing studies show that the emission intensity of GHGs and air pollutants varies greatly in different regions at various developmental stages, with significant spatial correlations and spatial agglomerations [46,47]. This study selects comprehensive scores of the co-benefits of carbon reduction and air pollution control as the core index and measures the global Moran index using Geoda software based on the adjacency weight matrix [48]. To further reveal the spatial distribution characteristics of the co-benefits of carbon reduction and air pollution control in the YRB, Geoda was used to draw the Moran’s scatter map. The display results of the global Moran index and Moran’s scatter diagram are presented in Table 3.

### 3.3. Analysis of Spatial Spillover Effects

The global Moran index of the co-benefits of carbon reduction and air pollution control in the YRB passed the significance test, indicating that it had a significant spatial dependence; this provides a reference for further revealing the spatial spillover effect. This study constructed the driving factor system and selected comprehensive scores of the co-benefits of carbon reduction and air pollution control as the dependent variable, while the independent variable was selected from the following aspects: (1) The level of economic development indicated the GDP. Economic growth will affect GHG emissions and air pollutants through scale, technology, and structural effects [49]. (2) The proportion of added value of secondary industry in GDP was selected as the industrial structure, and the adjustment of industrial structure affected GHG emissions and air pollutants [50]. (3) The level of urbanization indicated the urbanization rate. In the early stage of urbanization, the production operation may cause high carbonization of people’s lifestyles. The latter stage of urbanization can also reduce carbon and air pollutant emissions through scale and agglomeration effects [51]. (4) The scientific and technological innovation level was determined by the research and experimental development funding intensity. The progress of production technology and the upgrading of environmental protection technology can effectively control GHG emissions and air pollutants [52]. (5) The level of opening up was expressed as the total volume of import and export trade. The transfer of GHGs caused by foreign trade directly affects domestic GHG emissions [53]. (6) The level of foreign investment was expressed as the actual amount of foreign investment. The pollution halo or pollution refuge effect of foreign investment needs to be further tested [54]. (7) The average number of years of education per capita can directly reflect the education level of a region. The cultivation of high-quality talent will enhance people’s awareness of environmental protection, promote technological innovation, and indirectly reduce GHG emissions and air pollutants [55]. (8) The intensity of environmental regulation selects the proportion of environmental pollution control investment in GDP. Environmental pollution control can effectively solve environmental pollution problems. Relevant data were obtained from the China Statistical Yearbook, provincial statistical yearbooks, and the China Environmental Statistical Yearbook.

In this study, Stata software was used for the regression analysis. When spatial variables were not considered, the Hausman test on the panel data showed that the Hausman statistic was significantly positive, such that the fixed effect model was more suitable. Based on the fixed effect results, this study used the SAC model to explore the spatial spillover effect after adding spatial variables. The empirical estimation results are listed in Table 4.

## 4. Results and Discussion

### 4.1. Co-Benefits of Carbon Reduction and Air Pollution Control

According to the dynamic evolution of the comprehensive scores of the co-benefits of carbon reduction and air pollution control in nine provinces in the YRB from 2006 to 2018, Figure 1 shows that the co-benefits of all provinces improved significantly. Among these, the co-benefits of Shandong Province increased significantly (45.7%), from 64.44 in 2006 to 94.22 in 2018. In 2018, the population and total economy of Shandong Province accounted for 52.2% of the total of the YRB, with high co-benefits due to the fact of its higher foreign trade import and export at the mouth of the Yellow River and its emphasis on ecological protection, talent cultivation, and efficient utilization of resources [56]. However, the co-benefits of Qinghai Province were the lowest over the years, accounting for only 43.6% of Shandong Province’s numbers in 2018. The co-benefits of Gansu Province showed the least improvement, and first experienced an increase and then a decrease. The co-benefits of Shaanxi Province presented an “inverted U” type from 2007 to 2009, and the peak value in 2008 was inseparable from the promulgation and implementation of the “Shaanxi Qinling Ecological and Environmental Protection Regulations” [57]. In terms of the different stages, the overall score of the co-benefits of carbon reduction and air pollution control in the YRB showed a steady growth trend. Over the past decade, the provinces in the YRB paid increasing attention to green and low-carbon development, and this effect was very obvious.

**Figure 2:**
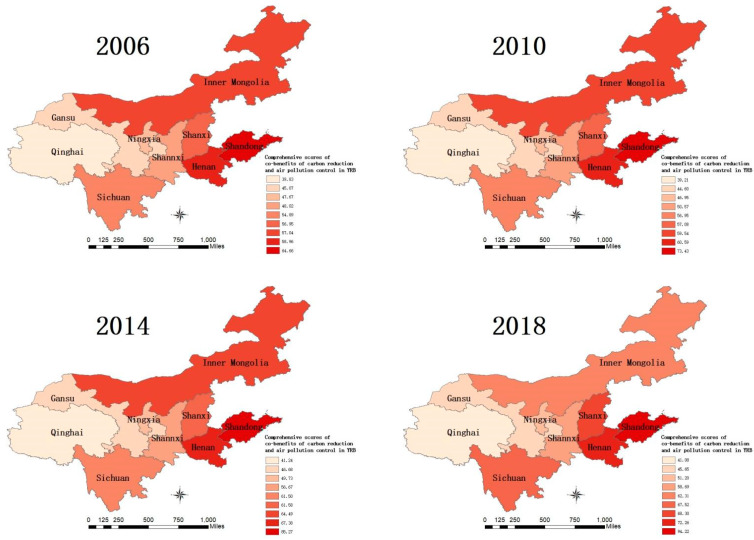
Charts of the co-benefits of carbon reduction and air pollution control in the YRB in 2006, 2010, 2014, and 2018.

From the perspective of the spatial differentiation of the comprehensive scores of the co-benefits of carbon reduction and air pollution control in nine provinces in the YRB, Figure 2 shows that the co-benefits of the provinces were significantly different, and the absolute difference expanded each year, from 24 to 53 from 2006 to 2018. In terms of the average value, the provinces in the YRB showed an increasing trend every year, but the average value of the eastern provinces was significantly higher than that of the central and western provinces while that of central provinces was higher than that of western provinces. Although the Inner Mongolia Autonomous Region is located in the economically underdeveloped northern region, its co-benefit ranking was always in the middle and upper reaches with an increase of 9.2% from 2006 to 2018. However, the region is a pioneer in the practical applications of the concept of green and low-carbon development [58]. The co-benefits of the central provinces were relatively stable, which was closely related to the fact that Shanxi Province and Henan Province are resource-intensive provinces. In a strongly developing economy, better consideration should be given to ecological benefits [59]. In the western provinces, the co-benefits of Sichuan Province were higher than those of other provinces and cities, and there was a significant improvement from 2006 to 2018. In addition, other provinces in the western region were affected by the relative weakness of economic development.

From the inter-regional convergence of the comprehensive scores of the co-benefits of carbon reduction and air pollution control in the nine provinces of the YRB, the standard deviation of the co-benefits increased each year from 2006 to 2018. It can be seen that due to the differences in economic and social development, the gap between provinces was gradually widening. In terms of region, the standard deviation of the eastern region was higher than that of the western and central regions. The central region presented better implementation of carbon reduction and air pollution control, and environmentally sustainable development was relatively stable, which was closely related to the pollution control policies that have been implemented in recent years [60]. In terms of the coefficient of variation, the YRB, as a whole, increased from 0.14 in 2006 to 0.24 in 2018, showing a divergent trend. In summary, from 2006 to 2018, the co-benefits of carbon reduction and air pollution control in the provinces of the YRB increased steadily each year. In terms of region, the co-benefits of the eastern provinces were higher than those of the central and western provinces, and the overall difference between the central and western provinces was small, showing the characteristic of the central and western regions lagging behind the eastern region, further exposing the imbalance of environmentally sustainable development in the YRB.

### 4.2. Spatial Correlation Analysis

According to Table 3, all the Moran’s I values were significantly positive, which indicates that the co-benefits of carbon reduction and air pollution control in the YRB had significant spatial positive correlation. The provinces with high or low co-benefits were significantly spatially concentrated; that is, they presented the characteristics of a significant “Matthew effect” [61]. The emission and flow of GHGs and air pollutants are strongly affected by spatial factors. However, ignoring spatial factors leads to deviations between the model estimates and empirical conclusions. From the perspective of the development process, the emission levels of GHGs and air pollutants in the YRB from 2006 to 2018 showed a fluctuating “up–down–up” development trend. As a result, the spatial autocorrelation also showed an upward and downward fluctuation trend; specifically, the spatial correlation showed an upward trend from 2006 to 2008, reaching the highest in 2008, showing a downward trend from 2008 to 2012, and showing an upward trend from 2012 to 2018.

Table 3 depicts the specific internal structure of the spatial agglomeration of the co-benefits of carbon reduction and air pollution control in the YRB. The empirical test indicates that only Henan Province presented H–H agglomeration, Gansu and Qinghai belonged to L–L agglomeration, no provinces had L–H agglomeration, and Sichuan Province presented H–L agglomeration over the years. The H–H agglomeration only appeared in Henan Province in 2017. As a province with a large population, Henan Province has a strong labor force, and its economic development has achieved remarkable results in recent years. In pursuit of regional economic growth, environmental regulations are followed, with limited environmental problems. Gansu and Qinghai showed L–L agglomerations. These two provinces are constrained by physical and geographical conditions, with a small population and sparse distribution, coupled with traffic constraints, relatively lower level of economy, and fewer environmental problems. The economic development level of Sichuan Province was relatively high in H–L agglomeration types. In recent years, it has become the strategic highland of economic growth in the western region and has obvious advantages in attracting high-tech industries and talent cultivation. Therefore, it may promote the development of high energy consumption, high-pollution, and high-emission enterprises in surrounding areas and cause environmental problems [62].

### 4.3. Analysis of Spatial Spillover Effects

Table 4 shows that in the fixed effect model, GDP, the proportion of the added value of the secondary industry in GDP, the actual utilization of foreign capital, and the year of education per capita passed the significance test. However, the urbanization rate, the investment intensity into research and development funds, the total amount of import and export trade, and the proportion of environmental pollution control investment in GDP did not pass the significance test. This represents the level of urbanization, the level of scientific and technological innovation, the level of opening up, and environmental regulations and indicates that these factors have little effect on the co-benefits. However, after considering the spatial variables, the urbanization rate and the proportion of environmental pollution control investment in GDP passed the significance test. This indicates that the level of urbanization and environmental regulation can still play an effective role in the co-benefits of carbon reduction and air pollution control. To reveal the degree of the spatial spillover effect, direct effect, indirect effect, and total effect were calculated by effect decomposition. The results are presented in Table 5. Among these effects, the indirect effect is the spatial spillover effect, which reflects the average impact of the driving factors of the province on the co-benefits of carbon reduction and air pollution control in neighboring provinces.

Table 5 shows that economic development level, urbanization level, foreign investment level, education level, and environmental regulation intensity had certain significance in the indirect effect. In other words, these five driving factors indirectly had a significant impact on the co-benefits of carbon reduction and air pollution control through the spatial spillover effect. In addition, the level of scientific and technological innovation and the level of opening up were not significant in the three effects, and the spatial effects representing the three driving factors were not obvious.

The level of economic development had a significant positive effect on the co-benefits of carbon reduction and air pollution control in neighboring provinces, which indicates that an improvement in economic development in this region will lead to an increase in co-benefits in neighboring regions. With the economic growth of the YRB, the increase in people’s income, and the increase in public attention to environmental quality, demand has gradually shifted from simple materials to considering the quality of material and ecological environment, thus driving the coordinated emission reduction of GHGs and air pollutants [63]. The agglomeration effect and diffusion effect of economic development level were the endogenous driving forces for the sustainable coordinated development of the environment in the YRB as well as a fundamental path to enhance the co-benefits of carbon reduction and air pollution control [64].

The level of urbanization had a significant negative effect on the co-benefits of carbon reduction and air pollution control in neighboring provinces, which indicates that the development of urbanization will reduce the co-benefits of neighboring provinces, and the spillover effect will gradually decrease with the increase in geographical distance. As many provinces in the YRB are still in the transition period from industrial society to post-industrial society, the urbanization process is very rapid. The urbanization rate increased by nearly 41.2%, from 39.8% in 2006 to 56.1% in 2018. The spillover effect of urbanization led to large-scale environmental pollution by driving GHG emissions and air pollutants and showed strong spatial mobility [65]. In addition, the high concentration of population and industry and the extensive state of spatial expansion caused by urbanization lead to a serious load on the resources and environmental carrying capacity, which then reduce the co-benefits of adjacent areas and hinder sustainable development of the environment in related areas.

The level of foreign investment had a significant negative effect on the co-benefits of carbon reduction and air pollution control in neighboring provinces, which indicates that the level of foreign investment will reduce the co-benefits of neighboring provinces. This is due to the development of the secondary industry in the YRB, and the relatively lower level of economic development and production technology. Foreign investment will lead to the transfer of high energy consumption, high-pollution, and high-emission enterprises to adjacent areas, that is, the pollution refuge effect, causing a large amount of air pollutant discharge, which is beyond the planned pollution control capacity of adjacent areas [66,67]. The extensive development mode of many provinces in the YRB also restrains technological progress, resulting in negative spillover effects on neighboring provinces.

Education level has a significant positive effect on the co-benefits of carbon reduction and air pollution control in neighboring provinces, which indicates that the improvement in the education level can effectively enhance the co-benefits of neighboring regions. The average number of years of education in Shanxi Province from 2006 to 2018 was 9.2 years. Many provinces in the YRB, such as Shanxi, Shaanxi, and Henan, have achieved a high degree of popularization of education. The average years of education in the YRB also increased from 7.8 years in 2006 to 9.0 years in 2018. The improvement in the education level can attract more high-quality talent, promote technological progress and industrial upgrading, and enhance the co-benefits of the region as well as neighboring provinces.

The intensity of environmental regulation had a significant positive effect on the co-benefits of carbon reduction and air pollution control in neighboring provinces, which indicates that environmental regulation can effectively promote the co-benefits of neighboring regions. Reasonable regional environmental regulation policies can effectively encourage enterprises to upgrade, improve production efficiency, and optimize resource allocation under the premise of energy conservation and environmental protection, to promote green development of the environment [68]. Considering the supply and marketing cooperation between enterprises in neighboring areas, investment in environmental pollution control will partially reduce the cost of emissions reduction and pollution control of local and neighboring provinces, thus promoting sustainable development of the environment.

## 5. Conclusions

“Ecological priority and green development” have become the inevitable path for the sustainable development of high-quality environments in the YRB. Considering the multiple backgrounds of the energy crisis, greenhouse effect, and air pollution, it is necessary to explore the spatiotemporal differentiation, spatial correlation, driving factors, and spillover effects of the co-benefits of carbon reduction and air pollution control in the YRB. Based on the framework of DPSIRM, this study constructed an evaluation index system and used the entropy weight method to assign objective weights to obtain the comprehensive scores of the co-benefits of carbon reduction and air pollution control in the YRB. In addition, the global Moran index and scatter plot were used to test the spatial correlation characteristics, and a generalized spatial econometric model was constructed to estimate the driving factors and spatial spillover effects. The results are as follows.

From 2006 to 2018, the co-benefits of carbon reduction and air pollution control in various provinces in the YRB increased steadily each year. Geographically, the co-benefits of eastern provinces were higher than those of the central and western provinces, showing that the co-benefits of the central and western provinces lagged behind those of the eastern provinces. The co-benefits of carbon reduction and air pollution control in the YRB had significant spatial positive correlation, which presented an “up–down–up” development trend. In 2017, Henan showed H–H agglomeration, Gansu and Qinghai presented L–L agglomerations, and Sichuan showcased spatial heterogeneity of co-benefits over the years. From the perspective of the spatial spillover test, the level of economic development, the level of education, and the intensity of environmental regulation had significant positive effects on neighboring provinces. Urbanization and foreign investment had significant negative effects on neighboring provinces. However, the level of scientific and technological innovation and the level of opening up did not pass the significance test; that is, the spatial spillover effect was not obvious.

Considering the above conclusions, this paper presents the following policy recommendations to promote the green and low-carbon sustainable development of the YRB: (1) Balanced development of the YRB should be promoted, focusing on the spatial spillover characteristics of the co-benefits of carbon reduction and air pollution control in the whole YRB region. The spatial transmission mechanism of GHG and air pollutant emissions and transfer should be clearly understood, and the emission reduction targets for carbon and air pollution that reasonably decompose and comprehensively coordinate also need to be evaluated in various provinces in the YRB. (2) There should be emphasis on promoting the transformation and upgrading of relevant industrial structures, strictly controlling and eliminating industries with high energy consumption, high pollution, and high emissions in the YRB; developing the modern services and high-tech industries; realizing the positive interaction between economic growth and collaborative emission reductions of carbon and air pollutants. (3) The industrial environment access system must be strictly implemented in the YRB, and the flow of foreign investment to high energy consuming, high pollution emitting, and low-efficiency industries should be restricted. Foreign investment needs to be encouraged in the field of green environmental protection. (4) The research and development of technologies for energy conservation and environmental protection need to be promoted, especially for clean energy and renewable energy technologies. The activities of exchanging and sharing the innovations and knowledge regarding green and low-carbon sustainable development should be encouraged in the YRB. (5) Additional policies can be designed to increase investment in education, attract high-quality talent, and promote effective collaboration between the YRB and other provinces and regions in the area of green and low-carbon sustainable development.

There remain several research problems that can be explored and discussed in future studies. The inter-regional transfer of polluting industries with high energy consumption, high pollution, and high emissions plays a dual role of angel and devil in the sustainable development of environment. The spillover effect of technological innovation undoubtedly also plays a significant role in the dissemination of green and low-carbon technologies, which can partially offset the effect of pollution refuge [69]. The level of technological innovation failed to pass the significance test in the spatial spillover effect test in this study because of the existence of this dual effect. To deal with this problem depends on the specific type of inter-regional transfer of industries, the stage of economic development, and the spatial structure of GHG and air pollutant emissions; all of these need to be further verified in future research. Due to the availability of the data for collection, the study region was selected at the provincial level in this work. However, it would be better if the data could be collected at the city level. These problems will be addressed in future research.

## Figures and Tables

**Figure 1:**
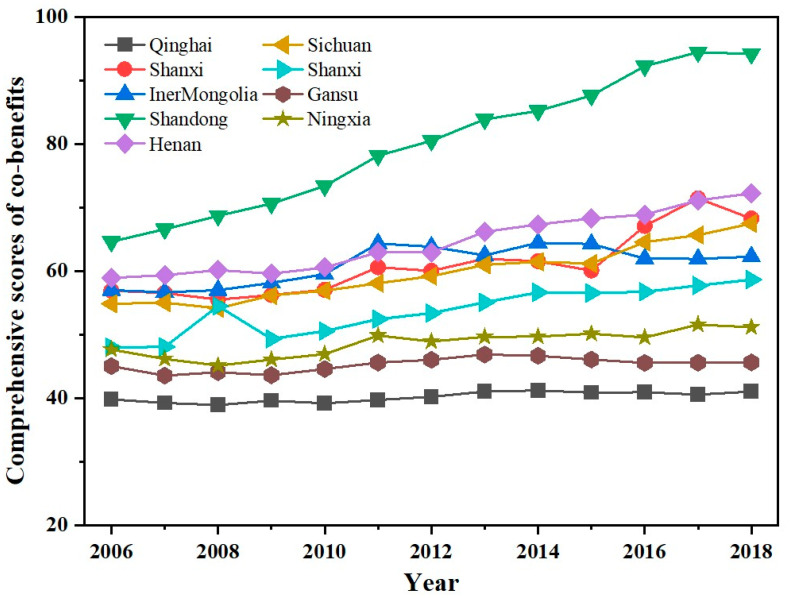
Line chart of the comprehensive scores of the co-benefits of carbon reduction and air pollution control in the YRB.

**Table 1:** Evaluation index system of the co-benefits of carbon reduction and air pollution control.

Frame Layer	Element Layer	Index Layer	Weight
Driving force (D)	Economic development level	GDP	0.072
		GDP per capita	0.032
	Industrial structure	Proportion of secondary industry to GDP	0.012
		Proportion of tertiary industry to GDP	0.024
	Population size	Permanent population	0.062
	Scientific and technological innovation level	Number of green technology patents authorized	0.127
Pressure (P)	Energy consumption intensity	Total energy consumption	0.045
		Energy consumption per 10,000 yuan GDP	0.040
State (S)	Emission level of air pollutants	Annual average concentration of PM_2.5_	0.039
		Total annual SO_2_ emissions	0.040
	Carbon emission level	Carbon emissions	0.044
		Per capita carbon emissions	0.041
		Carbon emissions per 10,000 yuan GDP	0.052
Impacts (I)	Air pollution	Air quality index	0.019
	Greenhouse effect	Annual average temperature	0.026
Response (R)	Government measures	Elasticity coefficient of energy consumption	0.042
		Green coverage of built-up area	0.013
	Environmental protection fund	Proportion of energy conservation and environmental protection expenditure in the total financial expenditure	0.030
		Operation cost of industrial waste gas treatment facilities	0.065
Management (M)	Environmental regulation	Total investment in environmental pollution control	0.063
		Treatment capacity of industrial waste gas treatment facilities	0.110

**Table 2:** Comprehensive scores of the co-benefits of carbon reduction and air pollution control in the YRB.

Region	2006	2007	2008	2009	2010	2011	2012	2013	2014	2015	2016	2017	2018
Qinghai	39.83	39.27	38.94	39.62	39.21	39.74	40.23	41.07	41.24	40.90	40.96	40.57	41.08
Shanxi	56.95	56.59	55.53	56.27	57.08	60.64	60.07	61.96	61.58	60.12	67.14	71.48	68.30
Inner Mongolia	57.04	56.70	57.04	58.16	59.54	64.37	63.87	62.51	64.49	64.30	62.00	61.95	62.31
Shandong	64.66	66.64	68.74	70.67	73.43	78.19	80.56	83.93	85.27	87.68	92.31	94.49	94.22
Henan	58.96	59.40	60.19	59.64	60.59	62.99	62.97	66.23	67.38	68.31	68.91	71.19	72.26
Sichuan	54.89	55.07	54.19	56.20	56.95	58.12	59.23	61.02	61.50	61.22	64.60	65.70	67.52
Shannxi	48.02	48.10	54.53	49.37	50.58	52.47	53.42	55.10	56.67	56.58	56.76	57.77	58.69
Gansu	45.07	43.57	44.11	43.63	44.60	45.62	46.06	46.90	46.68	46.10	45.57	45.60	45.65
Ningxia	47.68	46.20	45.17	46.09	46.95	49.88	48.96	49.67	49.73	50.17	49.59	51.61	51.20
AVG	52.57	52.39	53.16	53.29	54.32	56.89	57.26	58.71	59.39	59.49	60.87	62.26	62.36
SD	7.41	8.18	8.59	9.01	9.65	10.81	11.20	11.87	12.30	12.99	14.46	15.26	15.07
CV	0.14	0.16	0.16	0.17	0.18	0.19	0.20	0.20	0.21	0.22	0.24	0.25	0.24

**Table 3:** Global Moran index measurement results of the co-benefits of carbon reduction and air pollution control in the YRB.

Year	Moran’s I	Value Z	*p*-Value ^1^	High–High (H–H) Agglomeration	Low–Low (L–L) Agglomeration	Low–High (L–H) Agglomeration	High–Low (H–L) Agglomeration
2006	0.2892	1.7964	0.048	\	Gansu	\	Sichuan
2007	0.3131	1.9133	0.036	\	\	\	Sichuan
2008	0.3608	2.2042	0.022	\	Gansu, Qinghai	\	Sichuan
2009	0.2791	1.8129	0.046	\	\	\	Sichuan
2010	0.2697	1.8039	0.045	\	\	\	Sichuan
2011	0.2702	1.7961	0.049	\	\	\	Sichuan
2012	0.2460	1.7386	0.049	\	\	\	Sichuan
2013	0.3098	2.0870	0.016	\	\	\	Sichuan
2014	0.3089	2.0586	0.022	\	\	\	Sichuan
2015	0.3222	2.1603	0.016	\	\	\	Sichuan
2016	0.3081	2.1340	0.018	\	\	\	Sichuan
2017	0.3336	2.2255	0.016	Henan	Gansu	\	Sichuan
2018	0.3303	2.1971	0.014	\	\	\	Sichuan

^1^*p* < 0.05 indicates that the result was significant.

**Table 4:** Table of the empirical estimation results.

Index	Variable	Fixed Effect			SAC		
		Estimated Coefficient	t Value	*p* > |t| ^2^	Estimated Coefficient	z Value	*p* > |z|
Economic development level (ECOL)	The GDP	0.7701	11.75	0.000	0.6279	6.41	0.000
Industrial structure (INDS)	The proportion of added value of secondary industry in GDP	−0.0954	−4.06	0.000	−0.0543	−1.84	0.066
Urbanization level (URBL)	The urbanization rate	−0.0498	−1.07	0.287	−0.1356	−4.83	0.000
Scientific and technological innovation level (STIL)	The research and experimental development funding intensity	−0.0486	−0.79	0.429	−0.0565	−0.78	0.438
Opening up level (OPEL)	The total volume of import and export trade	0.0082	0.30	0.763	0.0171	1.18	0.238
Foreign investment level (FORL)	The actual amount of foreign investment	−0.1118	−3.23	0.002	−0.0762	−2.17	0.030
Education level (EDUL)	The average number of years of education per capita	0.0710	1.81	0.074	0.0562	1.81	0.070
Environmental regulation intensity (ENVI)	The proportion of environmental pollution control investment in GDP	0.0263	1.15	0.254	0.0381	2.35	0.019
Hausman		87.49	0.000				
cons		−0.0001	−0.00	1.000			
Rho		0.9419			0.4471	3.94	0.000
Number of samples		117			117		

^2^*p* > |t| < 0.1 indicates that the result was significant.

**Table 5:** Results of the classification of spatial spillover effects.

Index	Direct Effect	Indirect Effect	Total Effect
ECOL	0.6849 *** ^3^ (8.16)	0.4779 *** (2.97)	1.1628 *** (8.28)
INDS	−0.0605 * (−1.84)	−0.0407 (−1.46)	−0.1012 * (−1.80)
URBL	−0.1455 *** (−4.85)	−0.1012 *** (−2.66)	−0.2466 *** (−4.91)
STIL	−0.0630 (−0.78)	−0.0414 (0.64)	−0.1044 (−0.74)
OPEL	0.0196 (0.96)	0.0159 (0.80)	0.0355 (0.91)
FORL	−0.0825 ** (−2.34)	−0.0558 * (−1.89)	−0.1383 ** (−2.38)
EDUL	0.0613 * (1.86)	0.0384 * (1.85)	0.0997 ** (2.01)
ENVI	0.0406 ** (2.44)	0.0268 ** (2.08)	0.0674 *** (2.58)

^3^ *** Represents *p* > |t| < 0.01 and a 1% significant level; ** represents *p* > |t| < 0.05 and 5% significant level; * represents *p* > |t| < 0.1 and a 10% significant level. If there is no *, this means the variable was not significant.

## Data Availability

The data sets generated and analyzed in the current study are available in the “China Statistical Yearbook”, “China Environmental Statistical Yearbook”, and the published statistical yearbooks of the provinces in the Yellow River Basin.
